# Evolving Health Information–Seeking Behavior in the Context of Google AI Overviews, ChatGPT, and Alexa: Interview Study Using the Think-Aloud Protocol

**DOI:** 10.2196/79961

**Published:** 2025-10-07

**Authors:** Claire Wardle, Shaydanay Urbani, Eric Wang

**Affiliations:** 1 Department of Communication Cornell University Ithaca, NY United States; 2 School of Public Health Brown University Providence, RI United States

**Keywords:** artificial intelligence, large language models, ChatGPT, Google, Alexa, search engine, health information–seeking behavior, trust

## Abstract

**Background:**

Online health information seeking is undergoing a major shift with the advent of artificial intelligence (AI)–powered technologies such as voice assistants and large language models (LLMs). While existing health information–seeking behavior models have long explained how people find and evaluate health information, less is known about how users engage with these newer tools, particularly tools that provide “one” answer rather than the resources to investigate a number of different sources.

**Objective:**

This study aimed to explore how people use and perceive AI- and voice-assisted technologies when searching for health information and to evaluate whether these tools are reshaping traditional patterns of health information seeking and credibility assessment.

**Methods:**

We conducted in-depth qualitative research with 27 participants (ages 19-80 years) using a think-aloud protocol. Participants searched for health information across 3 platforms—Google, ChatGPT, and Alexa—while verbalizing their thought processes. Prompts included both a standardized hypothetical scenario and a personally relevant health query. Sessions were transcribed and analyzed using reflexive thematic analysis to identify patterns in search behavior, perceptions of trust and utility, and differences across platforms and user demographics.

**Results:**

Participants integrated AI tools into their broader search routines rather than using them in isolation. ChatGPT was valued for its clarity, speed, and ability to generate keywords or summarize complex topics, even by users skeptical of its accuracy. Trust and utility did not always align; participants often used ChatGPT despite concerns about sourcing and bias. Google’s AI Overviews were met with caution—participants frequently skipped them to review traditional search results. Alexa was viewed as convenient but limited, particularly for in-depth health queries. Platform choice was influenced by the seriousness of the health issue, context of use, and prior experience. One-third of participants were multilingual, and they identified challenges with voice recognition, cultural relevance, and data provenance. Overall, users exhibited sophisticated “mix-and-match” behaviors, drawing on multiple tools depending on context, urgency, and familiarity.

**Conclusions:**

The findings suggest the need for additional research into the ways in which search behavior in the era of AI- and voice-assisted technologies is becoming more dynamic and context-driven. While the sample size is small, participants in this study selectively engaged with AI- and voice-assisted tools based on perceived usefulness, not just trustworthiness, challenging assumptions that credibility is the primary driver of technology adoption. Findings highlight the need for digital health literacy efforts that help users evaluate both the capabilities and limitations of emerging tools. Given the rapid evolution of search technologies, longitudinal studies and real-time observation methods are essential for understanding how AI continues to reshape health information seeking.

## Introduction

### Overview

Health information–seeking behavior (HISB) is the term used to describe the ways in which people seek out and use information related to their health, including attempts to understand potential health risks, to learn how to prevent disease, and to seek help with an existing diagnosis. This literature has highlighted the broad appeal and versatility of search engines in meeting diverse information needs related to health. According to the Centers for Disease Control and Prevention’s National Center for Health Statistics, 58.5% of people in the United States reported searching the internet for health information in the previous 12 months [1]. For over a decade, surveys have demonstrated that search engines are the most common platform for online information seeking [2], and this is supported in studies with a range of people, from everyday users [3,4] to patients with chronic health conditions [5-7]. A reliance on search engines is particularly the case for populations less able to access a primary care provider [8-15].

While Google search remains the most common digital tool for searching health information, new technological developments are significantly changing the “search” landscape. Some of the most notable examples include the integration of Google’s large language model (LLM), Gemini, used to provide “AI Overviews” at the top of Google search results pages; stand-alone LLMs, such as OpenAI’s ChatGPT or Anthropic’s Claude; and the popularity of voice assistants, such as Alexa or Siri. As a result of these technological advances, there is an emerging field developing around artificial intelligence (AI)-HISB [16-18]. Much of this early research into the impact of LLMs has focused on “auditing” the quality of the results around health topics [19,20], comparing levels of trust between AI-powered health information and primary care providers [21-23] and investigating selective exposure via LLMs [24]. This study builds on this literature by exploring the ways in which people are adapting their behaviors to these new AI-powered technologies when undertaking HISB.

### Background

#### HISB and Evaluating Sources Pre-LLMs

An early, influential study into HISB focused on telephone calls to the Cancer Information Service in the 1980s, an important reminder of the ways in which people sought information before search engines [25]. The model of Freimuth et al [25] detailed six steps: (1) comparing to previous knowledge; (2) identifying goals and time available to reach them; (3) weighing costs vs benefits of active search; (4) undertaking the search, which varies in intensity and method; (5) evaluating the information gleaned; and (6) deciding whether the information is adequate. This model has influenced the study of HISB over the last 4 decades, with many studies examining the factors that impact these 6 steps. Two recent systematic reviews of online HISB [26,27] have demonstrated key influencing factors, including age, sex, income, employment status, literacy (or education) level, country of origin, place of residence, and caregiving role. The literature included in the systematic review of Jia et al [26] suggests a number of factors that encourage online health information seeking, including the existence of online communities, adequate privacy protections, real-time interactions, and archived health information formats. They also outline key barriers, including health literacy, access, information retrieval skills, and the presence of low-quality information, which significantly impact information-seeking experiences.

Concurrently, the question of how people make decisions around the trustworthiness of a health source has also received significant attention [28-34]. The source credibility theory [35] has been applied to studies comparing doctors to online health sources [36], credibility judgments on social media [37], and user-generated content related to health [38], demonstrating how people combine considerations around trustworthiness and perceived expertise. Research on online health communities shows that advice from similar peers, those who share similar interests or health conditions, is often valued over expert guidance, illustrating how homophily can outweigh expertise [39]. It has also been observed that web users struggle with “source blindness,” or the failure to process cues related to sources, in situations of “information context collapse”—when different types of content are placed in the same form and location [40,41].

More recent research has continued to investigate the mechanisms by which users evaluate trust in online health sources. In 2019, Sillence et al [42] built on earlier work from 2011 [43] to describe their “Revised Model of Trust,” identifying four trust factors: (1) personal experiences, (2) credibility and impartiality, (3) privacy, and (4) familiarity. In addition, a recent meta-analysis considered the wide range of variables that affect people’s online HISB and concluded that similar variables—utility, trust, and information quality—were the most influential factors [27].

As there are currently no population-wide studies on GenerativeAI literacy, it is impossible to know whether people understand how LLMs are designed, created, and work. This study offers some very preliminary insights into how people consider “sources” in the context of ChatGPT and Google’s AI Overviews.

#### The Use of AI-Powered Voice and Text Technologies for Search

While survey research has started to explore how many American people are using LLMs for answers to health queries [17,44], there is a growing body of both quantitative and qualitative literature attempting to understand the factors that are motivating the adoption of this new technology for HISB [18,45,46], highlighting that user-centric factors (perceived usefulness, convenience, trustworthiness, and AI experience and literacy) as well as technology-specific factors (information quality and ethical issues) are affecting decisions.

An important body of literature that provides a foundation for this is that which has interrogated the ways in which trust functions in terms of AI systems generally [47-50]. The research of Oh and Jung [47] highlights people’s likelihood to trust AI to perform a task that is considered mechanical, technical, or purely functional (eg, calculations, data processing, and routinized tasks) but less likely to trust AI when the task required cognitive skills (eg, complex reasoning or judgment), managerial skills, or social interactions (eg, understanding human nuance).

Another body of literature has explored trust in voice assistants in particular [51-54]. This literature has emphasized the importance of functionality-related factors (is it reliable, accurate, and secure?) as well as anthropomorphic characteristics (is it friendly, empathetic, etc?). These arguments are supported by studies focused on non–AI-powered chatbots available during the COVID-19 pandemic, which found that user trust in chatbot answers was affected by the branding of the chatbot provider, disclosure, and empathy [55], as well as cultural sensitivity and the quality of language [56]. Another study focused on a chatbot designed specifically for Black and Hispanic female population emphasized the importance of language and tone for building trust [57].

While these conversational approaches provide new opportunities for reaching audiences, it also introduces new complexities in terms of user trust and behavior. Research on older adults’ use of voice assistants for health information seeking reveals a complex trust dynamic: older adults may use voice assistants for confirming existing health knowledge but distrust the results, demonstrating a preference for more traditional information sources, such as direct communication with a medical professional [58]. Furthermore, the design of voice assistants can inadvertently perpetuate inequalities in information access, which we know has been a serious problem with all forms of new technological adoption [12]. For example, another study found that Black older adults often feel compelled to modify their speech patterns, engaging in a form of “digital code-switching” to obtain desired results from a Google Home device [59]. This finding highlights the need to critically examine how design choices in voice assistant technology influence how people use the technology and perceive the results [60].

#### LLMs

The emergence of LLMs such as ChatGPT has sparked both enthusiasm and apprehension about their potential to transform information seeking. The relatively short time frame since the launch of consumer-focused LLM technology (November 2022) means research is still somewhat limited. Early research identified 6 reasons why users would turn to ChatGPT: productivity, novelty, creative work, learning and development, entertainment, and social interaction and support [61]. While comprehensive research into the use of ChatGPT or other LLMs to seek out health information is not yet available, some scholars have argued that the technology has the potential to not only provide information in accessible formats but also to simplify complicated advice [62].

Two recent surveys provide a sense of how LLMs are being used to search for health information. One survey of US adults (n=2002) found that 32.6% of respondents used LLMs to answer health-related queries. Of these respondents, 62% stated that they still typically relied on search engines first, but 14% said that they used LLMs first. The survey also found that LLMs were perceived as less useful and less relevant than search engines but appeared more human than search and were seen as less biased [17]. Another survey of US respondents (n=2406) found that 21.5% respondents reported using ChatGPT for online health information seeking. ChatGPT users were younger than nonusers (aged 32.8 vs 39.1 years) with lower advanced degree attainment (BA or higher) and greater use of transient health care. Around 39.3% respondents reported using the platform for online health information seeking 2 to 3 times weekly or more, and most sought the tool to determine if a consultation was required or to explore alternative treatment. In total, 87.7% believed ChatGPT to be just as or more useful than other sources of online health information, and 81% believed it to be more useful than their doctor. About one-third of respondents requested a referral (n=184, 35.6%) or changed medications (n=160, 31%) based on the information received from ChatGPT [44].

Concerns remain about the ability of LLMs to consistently deliver accurate information. A 2023 study that directly compared ChatGPT and Google revealed that ChatGPT struggled with specific and nuanced queries, particularly in specialized fields like health care [63]. However, more recent research shows that LLMs are slightly outperforming the search engines when answering health-related queries [19]. The lack of transparency in how LLMs generate responses is a significant concern for some users, who liked the convenience of using ChatGPT for health information seeking but expressed reservations about its credibility [46].

One study focused specifically on trust in health information provided by Google and ChatGPT found that prior domain knowledge affected levels of trust as well as previous experience using the search agents [18]. The study also found that trust rankings for health information were marginally higher for ChatGPT than for Google. This trust level did not differ significantly when the category of health information that people were searching for changed. There was sometimes but not always a correlation between trust in the health information provided and trust generally in the platform. The study also highlighted something critical that was also observed in this study: many people consider online searching to be an iterative process involving multiple information sources and platforms.

### Study Objectives

The aim of this paper is to examine qualitatively whether and how AI-powered tools are impacting how people search for health information. The study explores how people are adapting to new affordances such as voice-activated prompts, bite-sized summaries, and conversational interfaces, which expand the options available when searching for health information. These tools, particularly those which emphasize automated summaries designed for speed and convenience, represent a marked shift from traditional search engine use, where finding information required typing queries, scanning results, and reading and cross-referencing multiple sources. However, the convenience of short summaries has also prompted concerns about potential biases and misinformation [64-66]. Are people aware of these trade-offs, and if so, how are they navigating them? The research was designed around 2 research questions (RQs):

- RQ1: How have voice- and AI-assisted search technologies impacted HISBs?
- RQ2: How have these technologies impacted the ways in which people make sense of the health information they encounter?

## Methods

### Study Design

We used a think-aloud protocol to structure observations with 27 participants in the summer and fall of 2024 as they searched for health-related information on different platforms, primarily Google, Alexa, and ChatGPT 3.5. On rare occasions, participants were asked to use a similar technology they were more comfortable with, for example, if they were sitting next to their Google Home device (instead of Alexa) or had ChatGPT 4 (instead of 3.5) downloaded onto their phone. The 3 platforms were chosen due to market share. According to Statcounter, 86% of the US market uses Google on desktop. (We had originally been interested in participants’ responses to AI-generated “snippets” at the top of the search results page, not realizing that AI Overviews powered by Google’s Gemini would be rolled out when we were collecting data.) Alexa is the most popular voice assistant (61% according to Statista), and ChatGPT is the most popular LLM according to Elon University’s Imagining the Digital Future Center survey conducted in January 2025 [67].

This method enabled the researchers to prompt the participants to provide further justification for an action, explain how a particular search made them feel, or explain whether a search result met a threshold for taking action. This method has shown to be useful in usability test settings in order to understand people’s behavior with particular technologies [68,69], including a previous iteration of a study observing and listening to people search for health information online [70]. Three researchers (CW, SU, and EW) interviewed 27 people in total. There were 4 phases to the study design.

Phase 1 was designed as a way to understand how people self-reported their search behavior before introducing any specific platforms. The researchers asked the participant to reflect on 2 prompts without doing any actual searching:

Prompt 1:

Imagine that a relative comes to you and tells you that they have heard that a plant called “St. John’s wort” is effective at treating depression. Describe how you would go about finding out whether this is true.

Prompt 2:

Can you think of a question about your health and wellbeing that you have needed to research in the last few weeks? We are using a broad definition of health and wellbeing; for example, any question you had related to healthcare, food, housing, employment, education, or voting. How did you go about looking for that information?

Having the same prompt (prompt 1) for all participants was designed to compare and contrast behaviors. The topic of St John’s wort as a treatment for depression was selected because it is an example of “unsettled science” where there was a likelihood that there might be contradictory evidence produced by different search technologies. One study on consumer use of St John’s wort found that 74% of survey respondents admitted that they were taking the drug without seeking medical advice [71], suggesting some evidence for people seeking other forms of health information before making the decision to use the “drug.”

Prompt 2 was designed to give participants an opportunity to search on topics that were immediately relevant to their lives. This design choice was more important that we had expected. Much of the most descriptive and passionate responses took place during these portions of the interviews, giving us a window into the challenges and nuances of searching for health information that actually mattered to the participants.

In phase 2, participants were asked to research the same 2 prompts—the question about whether St John’s wort is effective for treating depression as well as the personal health question that they provided—using a Google search. As they performed the search, participants were encouraged to narrate their experience out loud: what they were seeing, what they were clicking on or not clicking on and why, and what they thought of the information provided by the sources they did examine. Researchers prompted the participant to specifically comment on (1) whether they trusted the information and why and (2) whether they would “use” the information and how. While the participants talked about all of the elements they were seeing on the search results page, as researchers, we were focused on their reactions to the AI-powered Overviews at the top of the page.

In phase 3, participants repeated the search and narration process for both prompts using ChatGPT as a search platform. In phase 4, participants repeated the same process but using Amazon’s Alexa (either the machine, if one was available during the interview, or the smartphone app [provided by the researcher if the participant did not have it on their phone]).

The topics that participants provided for their personal health questions in prompt 2 varied widely in risk and urgency, providing a broad view into how different users adapt to platforms across different types of queries. Table 1 provides a complete list of these questions.

**Table 1:** Participant health concerns for query.

Participant code	Health concern
Participant 1	Mammograms for female participant with dense breast tissue: are they effective?
Participant 2	Prognosis for first year breast cancer: dealing with health anxiety of their partner
Participant 3	Friend with cannabinoid hyperemesis syndrome: causes and cures for vomiting
Participant 4	Plane crashes and DEI^a^: fact-checking alleged correlation
Participant 5	Looking for a dependable and economic car
Participant 6	How sugar affects your mood?
Participant 7	Chemicals in sunscreen: are they worse than sitting in the sun for a limited amount of time?
Participant 8	Restrictions after a hysterectomy: what are they?
Participant 9	Chicken nuggets: what they are made of and whether they are healthy?
Participant 10	Cough and difficulty breathing: looking for Chinese medicine treatment
Participant 11	Keeping their health insurance: concern that Medicaid will “run out”
Participant 12	90-year-old grandmother had eye surgery: whether doctor’s prescribed medications were all safe to take
Participant 13	COVID-19: treatment options for the variant they had
Participant 14	Bruising easily: what laboratories and tests they should have?
Participant 15	Antibiotics: should you take 2 after skipping?
Participant 16	Increasing insulin prices over time
Participant 17	Employment: looking for jobs for writers in New York City
Participant 18	Stung by jellyfish: treatment and whether hospital is necessary
Participant 19	Torn meniscus: surgery options and healing process
Participant 20	Fainting spells: why they were happening?
Participant 21	Scalp dryness: how to reduce?
Participant 22	Father with dementia: learning about it and how it affects the brain?
Participant 23	Spasm in their levator scapulae: how to improve their condition?
Participant 24	High number of hyaline casts in urine analysis: is this a concern?
Participant 25	Sleep issues and insomnia: strategies for fixing
Participant 26	STI^b^ testing in New York City: where to do it?
Participant 27	Statins: should you take them?

^a^DEI: diversity, equity, and inclusion.

^b^STI: sexually transmitted infection.

The interviews lasted between 25 and 60 minutes. Participants were paid US $25 each for their time. The participants undertook their searches on their phones or the researchers’ laptops or phones, and in the case of the latter, all searches were conducted using incognito mode (where possible). Their screen activity was not recorded, although participants were frequently reminded to explain what they were observing on their screens.

### Recruitment

Each researcher used convenience sampling to recruit 8-11 people based on “loose connections,” for example, an acquaintance from the dog park, a member of a dance club to which one researcher also belongs, and attendees of a community center for older people frequented by one of the researchers’ grandparents. The exclusion criteria included anyone younger than 18 years of age, anyone whose primary language was not spoken by the research team, anyone who was unable to meet in person, and anyone who did not use search engines to search for information. If someone had not used Alexa or ChatGPT, they could be included.

The recruitment was undertaken in 4 different locations on the east coast of the United States (Northern New Jersey; New York City; Ithaca, New York; and Providence, Rhode Island). Due to the different backgrounds of the 3 researchers, the result was a relatively diverse sample, ranging from 19 to 80 years of age, including people who identified as first- and second-generation immigrants from China, Mexico, and Syria; who spoke Chinese, Spanish, Turkish, and Arabic as their first languages; and who included a range of educational and socioeconomic backgrounds. The breakdown of participant demographics is described in Table 2.

**Table 2:** Participant demographic breakdown.

Characteristic			Participant, n (%)
**Sex**			
	Male	11 (41)	
	Female	15 (55)	
	Nonbinary	1 (4)	
**Age (years)**			
	18-24	3 (12)	
	25-34	10 (37)	
	35-44	7 (26)	
	45-54	2 (7)	
	55-64	3 (11)	
	65-74	0 (0)	
	75+	2 (7)	
**Languages spoken**			
	English only	17 (63)	
	Chinese only (Mandarin)	1 (4)	
	Chinese and English	4 (14)	
	Spanish and English	3 (11)	
	Arabic and English	1 (4)	
	Arabic and English and Turkish	1 (4)	
**Experience with LLMs^a^**			
	Had used before	17 (63)	
	Had not used before	10 (37)	
**Education**			
	High school	7 (26)	
	Undergraduate degree	12 (44)	
	Graduate degree	7 (26)	
	PhD or professional degree	1 (4)	

^a^LLM: large language model.

Participants had a mixed level of prior experience interacting with GenerativeAI. Some were already frequent users of ChatGPT for personal use or for work, some had heard of ChatGPT but had never used it, and a few had never heard of it. It is important to note that Google had also begun incorporating “AI Overviews” at the top of its search results shortly after the interviews began, and Meta’s integration of Meta AI into search bars across Instagram, Facebook, WhatsApp, and Messenger was also relatively new. For these reasons, the interviews provided a near real-time opportunity to observe how people were responding to and thinking about a new technology as it was rolled out.

In total, 74% (n=20) of the sample had at least an undergraduate degree, and the same percentage were younger than 45 years of age, reflecting a deliberate attempt to oversample people who we felt would be more familiar with these technologies (of the 10 people who had not used an LLM previously, 7 of them were older than 50 years of age). In addition, we oversampled people with university-level education due to research that has demonstrated people with lower levels of education are less likely to go online to seek health information [10,72]. These sampling decisions are supported by recent research that ChatGPT users were younger than nonusers (32.8 vs 39.1 years) with lower advanced degree attainment (BA or higher) [44].

### Analysis

The interviews resulted in 370 pages of transcripts, which were qualitatively analyzed by the 3 researchers who carried out the interviews. Using a reflexive thematic analysis approach, the researchers undertook the analysis in the 6 phases outlined by Braun and Clarke [73,74]. First, familiarization of the transcripts took place with the researchers spending additional time focused on reading and annotating the transcripts of the interviews that they did not facilitate themselves. The second phase involved the researchers individually generating codes using an inductive process. This was followed by a group session where these individual codes were compared and combined, resulting in the identification of 34 codes. The 3 researchers then applied those codes systematically to all transcripts. The next phases included constructing, reviewing, and defining themes, which the team undertook independently and then collaboratively. The codes were synthesized and aligned against the 2 RQs, ultimately highlighting that participants rarely differentiated between factors related to the process of searching and factors related to the process of judging the quality and utility of responses. In the reporting phase, as the analysis was written up, the findings were shared with some of the participants to identify any points that were unclear or failed to capture the complexity of their experiences.

### Ethical Considerations

Ethics approval for this study was obtained from Brown University’s Institutional Review Board on May 20, 2024 (#00000404). Written informed consent was obtained through a 2-page consent form, which outlined the processes for ensuring data protection and confidentiality and explained the right of participants to withdraw from the research at any time. The form also outlined the compensation that would be provided upon completion of the interview. All transcripts were anonymized, and a participant code was used throughout the analysis process, ensuring no connection between participants and data. All participants requested that any information related to the research be published anonymously.

## Results

### Observations Specifically Related to the 3 Technologies

#### Overview

Across the board, participants noted that all 3 technologies observed in this study—Google “AI Overviews,” Alexa, and ChatGPT—came with pros and cons. Participants adapted to new tools quickly and found ways to incorporate them into their search strategies even when they were skeptical of their trustworthiness. In general, people selectively deemed tools useful depending on the seriousness of their health question, the action they intend to take with the information, and their current circumstances. The following are some topline attitudes participants displayed toward the 3 technologies.

#### Google’s Gemini-Powered “AI Overviews”

Roughly one-third of participants commented on Google’s AI Overviews during the interview, and 5 participants explicitly stated that they skipped over that section of the search results. Even among those who read the overviews, most expressed skepticism and doubts about their trustworthiness, noting in particular their lack of sources.

I think I still don’t know it enough to trust it. Just like with ChatGPT, I was very hesitant in the beginning. And so, with the AI stuff in Google, I feel like I read it, but then I go down to the results and like, look through everything myself, because I just don’t know it enough to trust it.

Participant 6

Oh, the first thing Google gives me, an AI overview. It does actually describe the basics of what they [hyaline casts] are. But I see “AI overview” and I’m gonna scroll past, because this was the Google AI that was like, “you need to eat three rocks per day.”

Participant 24

No participant reported stopping their search at this material. Instead, they continued to scroll through results or look for sources they trusted.

#### Alexa

In total, 17 of the 27 (63%) participants noted that they would not typically use Alexa for the kinds of questions we were asking them to research. Broken links and misdirections to Amazon web pages also led several participants to give up their searches. A total of 8 of the 27 (30%) participants noted that short, voice-activated responses can be convenient for a low-stakes question or simple tasks like asking the weather. A few also deemed the responses satisfactory if they aligned with information they heard elsewhere.

#### ChatGPT

In total, 14 of the 27 participants indicated that they already use ChatGPT as part of their search and had opinions on how to use it most effectively. Of the participants who were not familiar with the technology, the majority adapted quickly to it: rewording their prompts, learning how to be more conversational with the tool, and experimenting with how to integrate it alongside more traditional search tools.

In total, 23 of the 27 (85%) participants reported favorably on some aspect of ChatGPT’s formatting or efficiency, noting, for example, that the platform was fast, organized, or easy to digest. Participants reported that ChatGPT was good at summarizing and described it as especially helpful for generating keywords they might not have thought of before or angles to search from on other platforms. Participants also appreciated that the platform allowed them to ask questions more precisely and that the conversational flow of the platform was responsive to how they worded their searches:

One of the reasons I’m using ChatGPT is because you can use [it] as a conversational [tool], so you can ask questions as [a] sequence from the answer given to you earlier. So I can use this as just [a] conversation with someone I’m talking [to], like a doctor, and ask questions: “Is there any medication,” “Do I need to see a doctor.”

Participant 13

Two-thirds of participants explicitly expressed concern about ChatGPT not citing sources. However, notably, even participants who were more skeptical of the platform’s trustworthiness liked the format:

I’m still skeptical 100%. But I just find it useful in terms of like, I got what I need. I got a good summary. Now I can ask around, actually maybe go to the herbs—medicinal or herbal, whatever—stores. Or like, I can look at JSTOR now for an article if I really want to know.

Participant 19

### AI Tools Within Broader Search Behaviors

The idea of AI tools as a “starting point” or point of comparison was a common framework for how participants said they would incorporate these tools into their broader search strategies. Once again, even skeptical users described using the tools in this way.

I would definitely use it as a starting point. If I wanted a quick summary, then that might influence how I’m going to make my research questions for Google, versus just throwing in something super generic and having to keep rephrasing my question.

Participant 3

I guess I trust it [ChatGPT] less than the NIH website, because again, I’m not seeing any sources. It’s very easy to read though. And it really, it really puts everything into a nice category. So it’s easy to digest. I would use it, again, as a starting point.

Participant 15

So my experience with ChatGPT is sometimes it’s very accurate. And sometimes it’s very wrong. And I don’t know until I read through the thing that I’ve asked it whether I can trust it. I guess I usually will Google the things it’s telling me to verify, to like fact-check.

Participant 6

In other words, most participants did not see AI tools as replacing deeper investigation, but as a tool for testing new ideas and validating them through subsequent searches.

### Factors Influencing Platform Choice

Participants described several contextual factors that played a role in which platforms they believed were useful. First was the severity of the health challenge: the more serious the concern, the more likely participants were to say that they would verify information from different sources or on different platforms.

I would use it [ChatGPT] to maybe copy and paste [some keywords] and like, look for a better source. It’s more concise, and you know, just popped right up for me as like a singular answer. Which might be effective for some things, but for questions of health care, I would say it’s less effective, because I would want a better range of answers.

Participant 20

Second was the intended use of the information: whether it was preparing for a doctor’s visit or talking to a family member, the intended use of the information factored into both how much they scrutinized the platform and their preference for format. Third was the circumstances they found themselves in. A small number of participants noted that Alexa and ChatGPT’s voice activation feature was helpful in situations where they were on the go, for example, when they could not type (such as driving), or when socializing with other people and typing might appear rude. Others noted that time-sensitivity played a role; for example, if they were doing a quick fact-check with a family member and they wanted that person to hear the answer in the moment. Finally, participants noted the role that their own knowledge and experience played. Familiarity and understanding of platforms shaped how participants interacted with them. Participants who were aware that Alexa was owned by Amazon demonstrated hesitancy about the company’s potential bias. Participants who demonstrated a basic understanding of LLMs were more skeptical of ChatGPT’s ability to accurately report sources when asked for them. One participant whose native language was Chinese noted that Alexa was less useful to them because it would not recognize their accent. In general, participants had different knowledge levels of the various tools observed, and there were plenty of misconceptions.

### Characteristics That Make People More Likely to “Use” Content From AI-Powered Technologies

Participants were more likely to deem information from AI tools useful if 1 of 3 conditions were met: if it aligned with something they saw or heard before, if it identified trustworthy sources, and if it helped shape subsequent searches.

Approximately one-half of the participants mentioned familiarity as a factor in trust because it aligned with something they had seen or heard before. To the extent that participants did say they trusted the information they gathered from Google Overviews, Alexa, and ChatGPT, it was often because the information repeated ideas from previous platforms they had worked with before or information they had heard from health professionals.

I do [trust it], because it’s echoing what I saw already. If I hadn’t seen that other information, I would be less inclined to go with this because I don’t know where it’s coming from.

Participant 25

I only trust it because it verifies some things that I have previously seen.

Participant 20

Participants had varying responses for what sources they believed to be trustworthy, but common themes included government websites, information from hospitals and academic institutions, and information that referenced studies. Almost all participants, however, noted that citing sources was important.

Trust and usefulness did not always go hand-in-hand. Many participants expressed skepticism about the trustworthiness of AI tools, and 15 of the 27 (56%) participants still reported using information they provided if that information helped power further investigation and point their searchers in the right direction.

I know that AI often produces incorrect results, and there’s no citations there. When I’ve used ChatGPT, or stuff like it in the past, it’s been helpful only when it sort of leads me to something I can look into further. But I never take what it says as the truth.

Participant 20

Would I just trust this answer? No. But this would be a really good start.

Participant 3

### Characteristics That Make People Less Likely to “Use” Content From AI-Powered Technologies

Participants mentioned 6 conditions as reasons why they were less likely to deem information from AI tools useful: if there were no sourcing or low-quality sources; if there was no human oversight; if they felt there were so kind of in-built bias; if the response was considered too brief; if the response was voice-activated; and if there was a broken or inaccessible user experience, meaning it was too difficult to navigate the technology.

The most frequently cited issue across platforms was the absence of sourcing or poor sources. Participants heavily criticized AI Overviews and ChatGPT for not providing sources. ChatGPT was described as a “void” (Participant 25) and a “black box” (Participant 24). Alexa was the one tool that usually provided sources, but 11 of the 27 (41%) participants found them to be low-quality.

Participants repeatedly voiced discomfort with the idea of machines generating health advice autonomously. Almost two-thirds of participants expressed that they like to be involved in the search process, check information for themselves, or at least know that a human has played a role in the generation of the information.

I’m still unsure about AI. Right. Just because it has to get its source of data somewhere. It performed no tests. It did no studies. It did not earn a PhD. It just pulled from sources.

Participant 4

I would Google “St. John’s wort depression.” And I would probably select one or two things from the first page of results. And usually, the first thing I click on would be some AI-generated bullshit. And I would read a little bit of that, and then I’d probably try to find a forum like a Reddit or something that seemed like it was real people talking about it.

Participant 20

So I’m a little wary though. I think the question for me is with something like, ChatGPT and all these things, it’s not that I don’t trust them. Because I know it’s just pulling from all the data that’s out there in the world. But the fact there hasn’t been a human involved to sort of edit somehow or analyze or confirm that it’s true, it’s a little worrisome.

Participant 7

Just over a third of participants expressed concerns about AI platforms containing different kinds of built-in bias, either toward the company, toward certain kinds of politics, or toward the user’s previous search history. For example, some users thought that AI Overviews might be influenced by sites they had visited before. Four participants were explicitly skeptical of Amazon’s influence on Alexa and voiced suspicions about whether the company was trying to steer them toward Amazon products or content:

The thing I don’t like about Alexa, and any of these, is they’re distributed by a company that is selling you something all the time. And I tend to not trust that.

Participant 2

Other participants described their concerns that ChatGPT might be too sycophantic:

I think it works best when you try to kind of separate your questions, because the AI has a tendency, where if you ask the question, and then ask another question that follows up with like a preconceived conclusion, it tends to bias itself towards the preconceived conclusion you have, because I found that ChatGPT really doesn’t like disagreeing with people

Participant 16

So, okay, we know that Google provides data relevant to things that you often search, right. That’s how the algorithm works. So if you look up ... “is climate impacting asthma levels?” [on ChatGPT] are you going to get a different result than if Cindy from the Republican side looks up? ... I think that matters to me, because I don’t want it to just tell me what I want to hear.

Participant 4

With Alexa and AI overviews, 11 of the 27 (41%) participants noted that the information they provided was either brief or too confident (ie, did not share caveats or opposing viewpoints) to be useful.

The majority of the native English-speaking participants ignored Alexa’s auditory responses in favor of reading the information on the screen. Just over half of the participants noted that the voice-activated apps often misunderstood their questions or complained about another aspect of the voice activation. Even one user who appeared to frequently use the voice activation setting on ChatGPT expressed concern about the parasocial relationship that they or their loved ones could develop with such a feature.

In total, 5 of 27 participants were frustrated by broken or misleading “learn more” links on the Alexa app, especially when they led to generic or Amazon-based web pages.

And then when I click for more information, I get a general page from Amazon saying “Ask Alexa medical questions.” So now I’m lost. I was compelled by the answer and then asking for more information, I’ve lost it. And I actually don’t know how to get back to it now.

Participant 20

Then it led me to Amazon, and what if I don’t use Amazon? So there’s this assumption of, okay, everybody uses Amazon. Well, I don’t. So this information is not accessible.

Participant 19

## Discussion

### Principal Findings

This research supports the argument that existing HISB models established before digital search technologies emerged are still applicable in an age of AI- and voice-assisted technologies. However, these AI tools have also introduced new affordances and layers of complexity. Conversational querying allows users to ask follow-up questions in real time, creating a dynamic where exploration and evaluation seem to be happening not just sequentially, as many HISB models suggest, but simultaneously. More recent perspectives discuss how trust is iterative in online health contexts, but AI tools seem to be making this overlap between exploration and evaluation central to the search process [42]. Because AI outputs are shaped by opaque models and hidden training data, users must evaluate not only the accuracy of the health information itself but also the reliability of the AI system that mediates it. The following are some specific issues related to AI search tools that require additional consideration.

### Utility Over Trust

Existing models emphasize credibility, trustworthiness, and usefulness as primary drivers of HISB [75-77]. This study suggests that perceived usefulness and convenience can sometimes outweigh trust, especially if users planned to verify outputs later or cross-compare on other platforms. Participants who were skeptical of ChatGPT nonetheless described how they would incorporate it in their search process, for example, using the platform to find keywords and then using those keywords to refine their Google searches, or bringing an AI-generated summary to a doctor’s appointment for further inquiry. Therefore, even when perceived trustworthiness and utility are at odds with each other, actual search behaviors tend to be layered and highly contextual. This is in line with other recent research on AI-HISB, which is starting to demonstrate that a person’s likelihood to use AI is not necessarily based on how much they trust the technology [16].

### Responding to Different Levels of AI Literacy

There were plenty of misconceptions present in our interview transcripts, from people believing Google could be edited, to others believing ChatGPT was a tool that just summarizes Google, to confusion about which companies own different technologies and how ownership impacted search results. While many participants discussed their strategies for evaluating the credibility of “traditional” search results from a search engine, only 5 of the 27 (19%) participants mentioned the ways in which “training data” can affect the results of LLMs or voice-assisted technologies and how that might impact whether or not they would judge the “search result” from these technologies as credible. These findings suggest a need for cross-generational educational programs to support people’s decision-making around their health when relying on these newer technologies.

Conversely, some participants demonstrated that they were evaluating a tool’s usefulness in nuanced ways, and it often led to sophisticated cross-platform search strategies or the reliance on AI-powered summaries in certain contexts (eg, if they had to email a paragraph summary to a relative before a medical appointment). Our oversampling of younger, technologically educated participants highlighted some of these strategies and is a reminder that when undertaking qualitative sampling strategies for similar research, it is important to identify participants who have low experience with new technologies and those who are more advanced. Both sets of results highlight the need for educators and health professionals to recognize and understand the different strategies being used by people using AI- and voice-assisted tools for health-related searches.

### Familiarity and “Echo Chambers”

Several participants admitted that they were more likely to “trust” information from Alexa or ChatGPT if they had heard the same information previously. Psychologists have described this phenomenon as the illusory truth effect, explaining that people are more likely to believe information they have heard before, even if that information is false [78-80]. Taking into account this known vulnerability in human cognition, this connection between familiarity and trust warrants additional investigation.

Additionally, participants in this study voiced concerns that LLMs were establishing echo chambers, some hypothesizing that these partial responses might be happening because of the tendency of LLMs to reinforce a user’s existing belief in order to “please” them and to keep them logged into the platform longer. Recent studies suggest that concerns about LLMs and sycophancy are warranted [81,82]. There is significant research on the ways in which algorithms produce search results that mirror people’s worldviews, but the majority of that work has been carried out on social networks [83,84]. More recent research on search engines suggests that differences in user queries are more likely to result in different search results rather than personalization [85], raising serious questions about the different responses that would be produced by different prompts for AI- and voice-assisted technologies. There are 3 areas where additional research is needed. First, consistently auditing the results produced by algorithmically driven voice-assisted technologies, such as Alexa, Google Home, and Siri, based on previous user search histories as well as purchasing habits. Second, while replicating LLM results is essentially impossible [86], additional attempts to audit LLM health-related results across different users, particularly those who have very different worldviews, would be valuable. Finally, users’ perceptions of echo chambers in AI search tools over time require examination.

### The Role of Humans in a World of Machine-Generated Information

Previous research on HISB has established that even though the vast majority of people report using search engines regularly, in survey responses, people still underline their preference to talk to a health professional [87]. Participants in our study underscored the desire to know that a human had helped shape and verify the information they were seeing, and some explained turning to YouTube, TikTok, and Instagram to “see” the person giving advice. In an age of AI-generated “slop,” the need to visualize a person could become even more important [88]. Alternatively, the ubiquity of AI- and voice-assisted technologies might lead to an acceptance that it will not be possible to know whether a human has played a role in checking the information, and different norms around wanting these features might emerge.

### The Multilingual Experience

For 9 of the 27 (33%) participants, English was their second or third language. Interviews with these participants raised a number of important insights that also require additional research with larger samples. Studies have documented the challenges of using US-owned technology for people who speak languages other than English [89-91], but the adoption of LLMs raises further questions. Evaluation datasets often privilege English, formal registers, and culturally specific norms, hiding failures in low-resource or marginalized language settings [92]. Models trained on English materials and then translated into other languages are vulnerable to mistakes and inherent biases [93]. However, our research also highlights the ways in which multilingual users move across languages and platforms, potentially exacerbating these issues. For example, one participant who is second-generation Chinese believed that searching using the Chinese language on these 3 technologies would yield answers based on knowledge from China when they needed US-specific information. Another Mandarin-speaking participant talked about how they moved seamlessly between different platforms and languages: “I usually type in the name of the medicine prescribed to me by my doctor into Google and look to see what it is used for, and then go to YouTube to find a similar Chinese drug so I can go and buy it” (Participant 20). Another underexplored issue, related specifically to LLMs and health information, is the ways in which LLM training data rooted in “Western medicine” might not satisfy users who are looking for answers drawing from different medical practices, for example, indigenous or “Eastern medicine” practices.

Furthermore, we know that accents can affect credibility judgments with voice assistants [94,95], and our multilingual participants raised the challenges of making voice-assisted technologies understand their prompts. For example, one Mandarin speaker asked the facilitator to repeat the question to Alexa because the bot would not understand their accent. As more people turn to voice activation when using LLMs, additional research will be needed to understand the experience of people for whom English is not their first language. Examining the ways in which people think about the limitations of the data upon which LLMs were trained around language, medical practices and voice recognition should be included in future research trajectories within the field of AI-HISB.

### Limitations

This study had several important limitations. First, our sample was small and generated via loose connections in personal networks. Second, our use of the think-aloud protocol to observe search behavior has the potential for producing the Hawthorne effect [63], in which participants change their behavior because they know they are being observed. Finally, this study was not longitudinal. It provides a snapshot of experiences with AI- and voice-assisted technologies, with a small sample of people, at one particular point in time. Notably, 10 of the 27 (37%) participants had never previously used a stand-alone LLM; therefore, we were able to observe their first impressions of using ChatGPT rather than discuss existing experiences or attitudes. Unfortunately, undertaking research on these types of technologies means that results quickly become out of date. In the context of this research, the functionality of Google’s AI Overviews and ChatGPT changed during the short time frame of the study. Google’s AI Overviews now appear in response to more search queries and are longer in length compared to the period when the interviews were being conducted. Additionally, both Google Overviews and ChatGPT now link out to sources. A majority of the participants had noted the absence of sources in both tools as a serious drawback, which suggests that interviews conducted today would likely sound very different in relation to this issue. While we are describing this issue as a limitation of our research, we also, as a wider field, need to grapple with the challenges of conducting research on technologies that are evolving at such speed. Authors need to focus on which of their findings are likely to withstand platform design changes and model updates and which will not. More research is also needed to explore whether users understand these design changes.

### Conclusions

While our sample cannot be used to generalize to a wider population, our research provides some insights into the ways in which some people are adapting to new AI- and voice-assisted technologies and integrating them into their health-seeking behaviors, mixing and matching rather than replacing older technologies altogether. The results suggest that people evaluate these technologies in nuanced ways, thinking about their utility in certain contexts even when they doubt their trustworthiness and credibility.

Notably, existing models for HISB remain relevant. For example, the 6 elements of HISB outlined by Freimuth et al [25] in 1989 appeared in all of the 27 conversations. For example, participants comparing results on ChatGPT with what they had previously found on Google (element 1); using voice-activated technologies when they were driving or needed a quick debunk to a claim someone is making at a party (element 2); deciding whether an overview on Alexa or ChatGPT is sufficient for a quick summary or whether they need to take the time to search themselves on Google (elements 3 and 4); cross-referencing results using different LLMs or asking for sources they can look up on Google Scholar or PubMed (element 5); and finally, deciding at different stages of the search process whether the information they gleaned is sufficient. For example, a ChatGPT or Google Gemini summary might be good enough to print out to take to a doctor’s appointment or to email to a friend rather than a deep dive search that they would do when investigating advice from a doctor after a visit (element 6).

While the strength of existing models in an age of AI- and voice-assisted technologies is important, the emphasis on utility over traditional ideas about trust is a potential factor that we think will continue to be relevant and will need to be continuously examined with interface changes or updates to AI models. Similarly, the ways in which LLMs might be creating echo chambers or reinforcing existing user beliefs should remain a key point of examination in any studies of AI-HISB. Finally, while it is tempting to assume that the reported desire to receive information from a human in the health context will stay constant, it is also possible that tolerance for information without a human source will slowly disappear over time.

As people begin to use AI- and voice-assisted technologies, large representative surveys help us to understand the scale of uptake and changing attitudes toward the technologies. However, these surveys should be combined with qualitative methodologies to understand how people are actually using these technologies and how they are making sense of the information they are encountering. Over-the-shoulder and think-aloud protocols enable researchers to watch and hear people explain their search behaviors in connection to search queries that they are personally invested in. Asking our participants to offer research topics based on their own interests and needs provided this study with rich detail that would have been lost if relying solely on identical prompts.

Based on these observations, we recommend that researchers design and implement longitudinal mixed methods studies in the emerging field of AI-HISB rather than snapshots of user behavior at one point in time and that researchers allow participants to explore their own health questions rather than standardized ones.

## Abbreviations

AI: artificial intelligence
HISB: health information–seeking behavior
LLM: large language model
RQ: research question

## Data Availability

The datasets generated or analyzed during this study are available from the corresponding author on reasonable request. The loose connections between participants and the researchers and the sensitive nature of personal health queries warrant not making the interview transcripts publicly available.
